# Supplementary material for: Severely Damaged Freeze-Injured Skeletal Muscle Reveals Functional Impairment, Inadequate Repair, and Opportunity for Human Stem Cell Application
Source: Biomedicines. 2023 Dec 21;12(1):30. doi: 10.3390/biomedicines12010030 (PMC10813063; doi:10.3390/biomedicines12010030)
Supplement: Supplementary file 1 [file biomedicines-12-00030-s001.zip › biomedicines-2760875-supplementary.pdf]

Supplementary Information

**Human Target gene**	**Primer sequence**	**Annealing temperature (C°)**
IDO	F 5’-TGCTAAAGGCGCTGTTGGAA-3’<br>R 5’-TACACCAGACCGTCTGATAG-3’	60
HGF	F 5’-CAATAGCATGTCAAGTGGAG-3’<br>R 5’-CTGTGTTCGTGTGGTATCAT -3’	60
GAPDH	F 5'-CATCATCTCTGCCCCCTCT-3'<br>R 5'-CAAAGTTGTCATGGATGACCT-3'	60
COX-2	QHsaCED0042341 BioRad	60
**Mouse Target gene**	**Primer sequence**	**Annealing temperature (C°)**
eMHC	F GCCTTGTGCTTTCCCAGAG<br>R CGTACACGGACTTGGAGAG	60
Pax7	F TGTGCCGATATCAGGAGACT<br>R GTCCTTCAGCAGCCGGTC	60
Myod	F CGCTCCAACTGCTCTGATG<br>R GCGCCGCCTCACTGTAGT	60
Myog	F-CCATCCAGTACATCGAGCG<br>R-TGGACTGCAGGAGGCGCT	60
Il-6	F ACCGCTATGAAGTTCCTCTC<br>R AGTAGGGAAGGCCGTGGTT	60
Tnf-α	F AACTTCGGGGTGATCGGTC<br>R AGGGTCTGGGCCATAGAAC	60
Ccl2	F CTGCTGCTACTCATTCACCA<br>R CCTTCTTGGGGTCAGCACA	60
Il-10	F AGTTTTACCTGGTAGAAGTGAT<br>R ATCACTCTTCACCTGCTCCA	60
Arg1	F GAAGAATGGAAGAGTCAGTGT<br>R CCATGCAGATTCCCAGAGC	60
CD206	F ATGAGGCTTCTCCTGCTTCT<br>R TTGCCGTCTGAACTGAGATG	60
Gapdh	F CACCACCAACTGCTTAGCC<br>R GGATGCAGGGATGATGTTCT	60

Table S1. Primers used for the detection of genes involved in muscle regeneration and inflammation process.

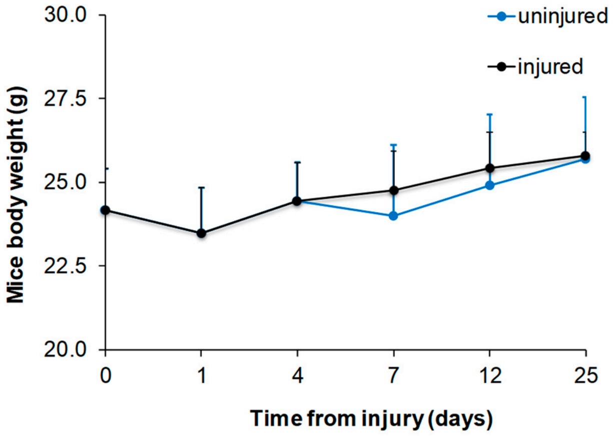

Figure S1 The injured mice did not demonstrate altered body weight compared to the uninjured mice at the experimental time points.

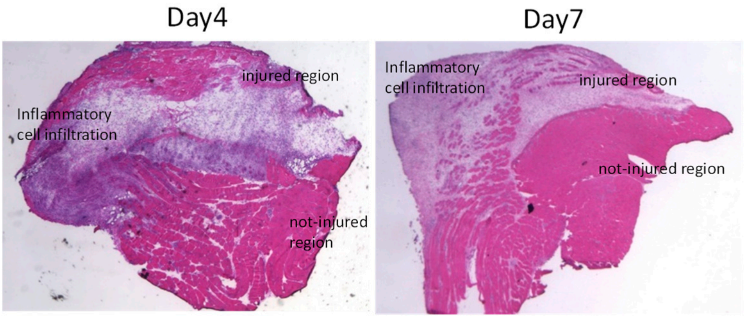

Figure S2 Qualitative histological observation of the entire frozen *biceps femoris* muscle showed the degree of injury and the substantial inflammatory infiltration. Representative images of hematoxylin and eosin-stained cross sections (10x).

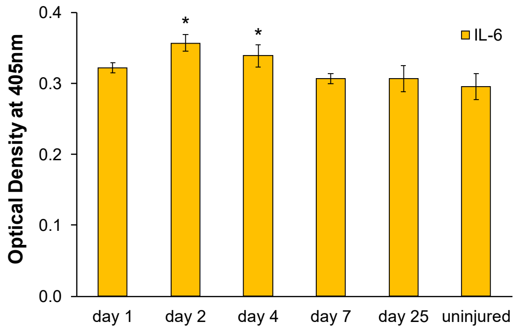

ng/ml ± SD	8.5±0.85	13.8±2	10.9±2.2	6.8±0.8	7±2	5.9±1.7
significance *vs.* control	*n.s.*	*P*=0.013	*P=0.045*	*n.s.*	*n.s.*	*n.s.*

Figure S3 FI generated systemic inflammatory IL-6 response. Proinflammatory cytokine IL-6 expression levels were investigated in uninjured control and injured mice after *biceps femoris* muscle damage. Detectable levels of IL-6 are shown in the table. Statistical significance was evaluated by one-way ANOVA by running a post hoc test. * $p < 0.05$ (one *vs.* uninjured.

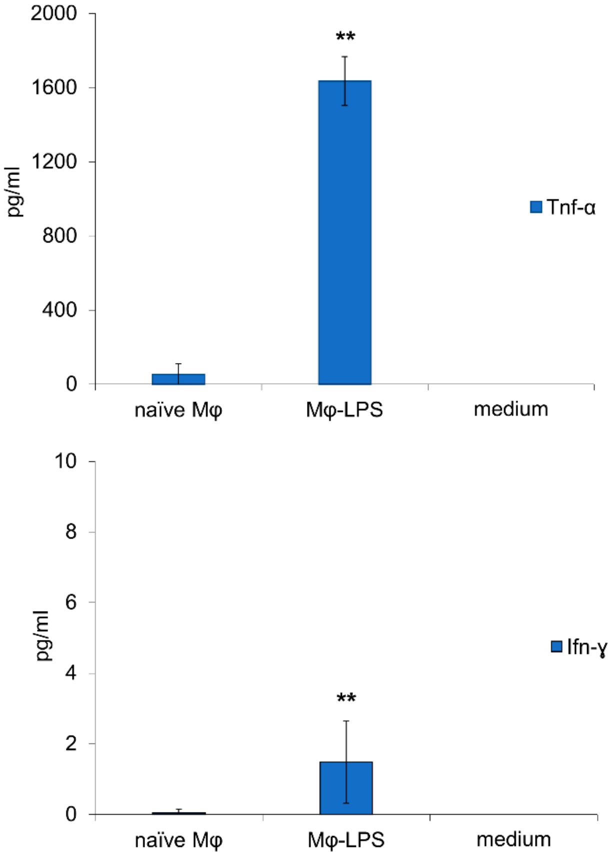

Figure S4 LPS treatment induced M0 RAW264.7 murine macrophages towards M1 phenotype transition. TNF-α and IFN- γ production was quantified in the supernatant of cultured naïve Raw 264-7 cells (naïve Mφ) and LPS-treated Raw 264-7 cells (Mφ-LPS) by ELISA assay. All data were expressed as mean ± SD (n = 4). Statistical significance was evaluated by Student's *t*-test.** p<0.001 *vs.* naïve Mφ

Supplementary Material and Methods

Serum Cytokines assay

Diluted sera 1:50 from uninjured control mice and injured mice, collected at 1, 2, 4, 7, and 25 days postinjury, were used for the detection of interleukin-6 (IL-6; mouse ELISA development kits; PeproTech® EC Ltd, UK) according to the manufacturer's instructions. Mouse sera and mouse recombinant standards were diluted in 1× PBS/0.05% Tween-20/0.1% BSA (Sigma-Aldrich®, Dorset, UK) and added to the microplates. Interleukin binding was detected in the biotin–avidin detection step, followed by chromogen 2,2′-azino-bis(3-ethylbenzothiazoline- 6-sulphonic acid) (Sigma-Aldrich®, Dorset, UK) incubation. Color development was monitored at 405 nm. The concentration of cytokines in the samples was determined from the standard curve, where detectable.

Supernatant Cytokines assay

Diluted supernatants 1:2 from cultured naïve Raw 264-7 cells (naïve Mφ) and LPS-treated Raw 264-7 cells (Mφ-LPS) were used for the detection of interferon γ (IFN-γ; Mouse IFN-γ ELISApro kit, MABTECH, AB Sweden) and tumor necrosis factor α (TNF-α mouse ELISA development kits; PeproTech® EC Ltd, UK) according to the manufacturer's

instructions. Color development was monitored at 450 nm (IFN-γ) and 405 nm (TNF-α). The concentration of cytokines in the samples was determined from the standard curve, where detectable.
